# Streptococcal Serine-Rich Repeat Proteins in Colonization and Disease

**DOI:** 10.3389/fmicb.2020.593356

**Published:** 2020-10-30

**Authors:** Jia Mun Chan, Andrea Gori, Angela H. Nobbs, Robert S. Heyderman

**Affiliations:** ^1^NIHR Mucosal Pathogens Research Unit, Division of Infection and Immunity, University College London, London, United Kingdom; ^2^Bristol Dental School, University of Bristol, Bristol, United Kingdom

**Keywords:** bacterial glycoproteins, *Streptococcus pneumoniae*, *Streptococcus agalactiae*, serine-rich repeat proteins, pathogenesis, colonization

## Abstract

Glycosylation of proteins, previously thought to be absent in prokaryotes, is increasingly recognized as important for both bacterial colonization and pathogenesis. For mucosal pathobionts, glycoproteins that function as cell wall-associated adhesins facilitate interactions with mucosal surfaces, permitting persistent adherence, invasion of deeper tissues and transition to disease. This is exemplified by *Streptococcus pneumoniae* and *Streptococcus agalactiae*, which can switch from being relatively harmless members of the mucosal tract microbiota to bona fide pathogens that cause life-threatening diseases. As part of their armamentarium of virulence factors, streptococci encode a family of large, glycosylated serine-rich repeat proteins (SRRPs) that facilitate binding to various tissue types and extracellular matrix proteins. This minireview focuses on the roles of *S. pneumoniae* and *S. agalactiae* SRRPs in persistent colonization and the transition to disease. The potential of utilizing SRRPs as vaccine targets will also be discussed.

## Introduction

It is well-established that glycosylation of eukaryotic proteins is important for regulating cellular processes, including receptor signaling and inflammation (Reily et al., 2019). In contrast, prokaryotic protein glycosylation was thought to be rare until the discovery of general *N-*linked and *O-*linked protein glycosylation pathways in *Campylobacter jejuni, Bacteroides fragilis*, and *Burkholderia cenocepacia*, revealing the role of glycoproteins in bacterial physiology and pathogenesis (Szymanski et al., 1999; Coyne et al., 2013; Mohamed et al., 2019). Many bacterial glycoproteins identified thus far are surface proteins that mediate host-microbe interactions and motility (Tytgat and Lebeer, 2014; Tan et al., 2015; Valguarnera et al., 2016; Eichler and Koomey, 2017; Bhat et al., 2019).

Members of the Gram-positive genera *Streptococcus*, *Staphylococcus*, and *Lactobacillus* express a family of large, glycosylated serine-rich repeat proteins (SRRPs) (Latousakis et al., 2020). They were first characterized as fimbrial-like or platelet-binding proteins in *Streptococcus parasanguinis, Streptococcus cristatus, Streptococcus gordonii*, and *Staphylococcus aureus* (Correia et al., 1997; Wu et al., 1998; Takahashi et al., 2002; Handley et al., 2005; Siboo et al., 2005). SRRPs mediate adhesion to sialic acid, fibrinogen, keratin or as yet unidentified molecules on other bacteria in a strain- or species-dependent manner (Deng et al., 2014; Six et al., 2015; Bensing et al., 2019; Latousakis et al., 2019). The versatility in binding partners suggests that SRRPs facilitate colonization of multiple and diverse niches, which in the case of oral streptococci permits these bacteria to persist in the mouth and to form infective vegetations on damaged heart valves (Yumoto et al., 2019).

*Streptococcus pneumoniae* and *S. agalactiae* (Group B *Streptococcus*, GBS) are important pathobionts of mucosal surfaces that disproportionately affect young children, the immunocompromised and the elderly. Studies estimate that pneumococci and GBS were respectively responsible for the death of around 300,000 and 90,000 children under 5 in 2015 (Seale et al., 2017; Wahl et al., 2018). Despite their notoriety as deadly pathogens, they are commonly found as asymptomatic colonizers of human mucosal surfaces, and may also cause milder diseases such as otitis media (*S. pneumoniae*) or urinary tract infections *(S. agalactiae)* (Feldman and Anderson, 2019; McLaughlin et al., 2020). Both *S. pneumoniae* and *S. agalactiae* have large accessory genomes, leading to considerable variation in pathogenic potential between serotypes and sequence types (Hiller and Sá-Leão, 2018; Chen, 2019). Genes encoding SRRPs are part of the accessory genome of both pneumococci and *S. agalactiae* (Tettelin et al., 2005; Gámez et al., 2018).

Research on SRRPs thus far has predominantly focused on oral streptococci, where these glycoproteins promote biofilm formation, intra- and interspecies aggregation and development of infective endocarditis (Zhou and Wu, 2009; Lizcano et al., 2012; Zhu et al., 2015; Zhu and Wu, 2016; Latousakis and Juge, 2018; Latousakis et al., 2020). However, there is growing evidence that SRRPs in *S. pneumoniae* and *S. agalactiae* may be important for the transition from asymptomatic carriage to disease. This minireview will focus on the functions of these SRRPs in colonization and disease. We will draw on evidence from studies of oral streptococci and closely related bacteria to compare and contrast the diversity, biogenesis and functions of SRRPs in streptococcal biology. Finally, we will discuss the potential of using SRRPs as vaccine targets for *S. pneumoniae* and *S. agalactiae*.

## Architecture of Streptococcal SRRPs

**FIGURE 1:**
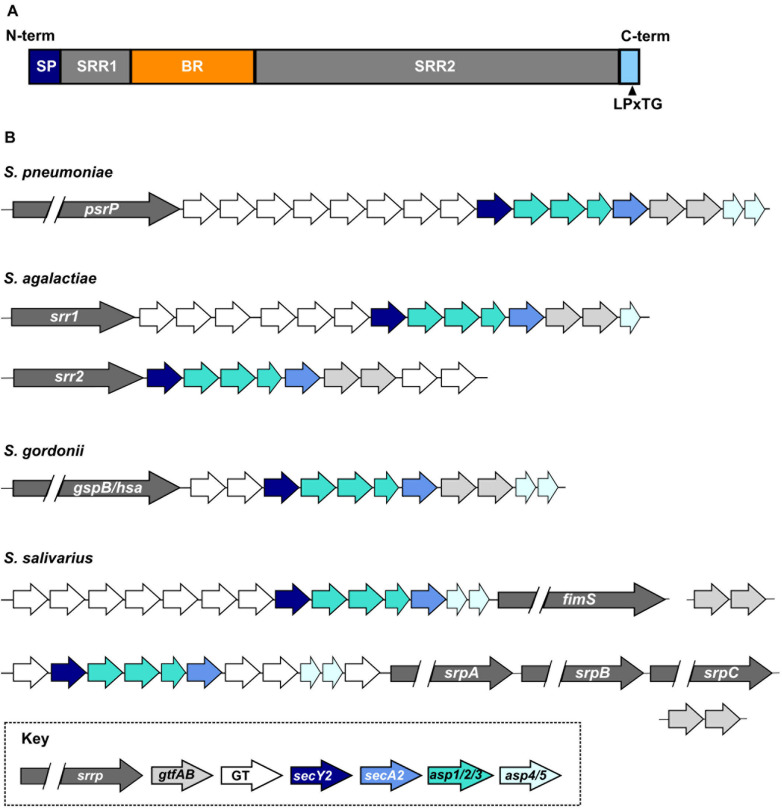
Organization and diversity of streptococcal SRRPs. **(A)** General architecture of the SRRP polypeptide. SP, signal peptide; SRR1/2, serine-rich repeat regions 1 or 2; BR, non-repeat binding region; LPxTG, sortase dependent cell-wall anchor signal. **(B)** Diversity of the SRRP glycosylation loci encoded by a subset of streptococcal species. *S. pneumoniae* carries only one SRRP encoding allele. *S. agalactiae* and *S. gordonii* encode two major SRRP alleles; the glycosylation island associated with each allele differs in *S. agalactiae* but not in *S. gordonii*. *S. salivarius* encodes two major SRRP islands that are associated with one or three SRRP alleles. Homologs of the *S. salivarius gtfA* and *gtfB* are encoded elsewhere on the chromosome. GT refers to accessory glycosyltransferases.

Serine-rich repeat proteins are characterized by the presence of (i) an extended N-terminal signal sequence, which facilitates transport of the protein through an accessory secretion system; (ii) two highly glycosylated serine-rich repeat regions (SRR); (iii) at least one non-repeat binding region (BR); and (iv) a C-terminal cell wall anchoring domain carrying a LPxTG motif (Figure 1A). Despite architectural conservation, SRRPs share little sequence homology especially in the sequences and lengths of the SRR and BR domains, resulting in significant variation in protein size and binding partners (Zhou and Wu, 2009; Lizcano et al., 2012; Bensing et al., 2019). The streptococcal SRRP preprotein ranges in size from 970 amino acids (*S. agalactiae* Srr1) to over 5000 amino acids (*Streptococcus oralis* subsp. *dentisani* FapC) (Seifert et al., 2006; Ronis et al., 2019).

Protein crystallography and electron microscopy show that SRRPs have a fimbriae-like structure, where the second SRR domain (SRR2) forms an extended stalk structure that projects the globular BR from the cell surface (Wu et al., 1998; Handley et al., 2005; Ramboarina et al., 2010; Lizcano et al., 2012; Six et al., 2015). Expression of a truncated version of the pneumococcal SRRP, PsrP, rescues binding defects of a *psrP* mutation in capsule-null, but not in encapsulated *S. pneumoniae* (Shivshankar et al., 2009). Thus, the length of the SRR2 region is hypothesized to have adapted to extend the BR beyond the polysaccharide capsule in encapsulated streptococci (Shivshankar et al., 2009; Lizcano et al., 2012). The SRRP BRs of *S. parasanguinis* and the gut bacterium *Lactobacillus reuteri* assume different conformations in low and high pH, thereby altering binding affinity (Garnett et al., 2012; Sequeira et al., 2018). It is not known if similar conformation shifts occur with other streptococcal SRRPs.

### Heterogeneity Of Streptococcal SRRPs Encoded On Glycosylation Islands

Streptococcal SRRPs are encoded on putative genomic islands, which we will refer to as glycosylation islands (Takamatsu et al., 2004b). The minimal SRRP locus includes eight genes encoding (i) the SRRP; (ii) an accessory secretion system (*secA2, secY2, asp1, asp2, asp3*; Asp proteins are also known as Gap); and (iii) core glycosyltransferases (known in different species as *gtfAB*, *gtf1/gft2*, and *gtfE/F*) (Figure 1B; Zhou and Wu, 2009). Frequently, glycosylation islands include additional accessory genes. While all genes within the minimal locus are typically found on the island, *Streptococcus salivarius gtfAB* homologs are encoded elsewhere on the chromosome (Pombert et al., 2008; Couvigny et al., 2017). Mutations of any gene in the minimal locus typically impairs SRRP function and cell surface presentation (Takamatsu et al., 2004b; Wu et al., 2007; Mistou et al., 2009; Lizcano et al., 2017).

Members of the genus *Staphylococcus* encode the minimal SRRP locus consisting only of the eight genes described above (Siboo et al., 2005). In contrast, streptococcal SRRP loci may harbor up to eight additional genes encoding accessory glycosyltransferases (GTs) (Figure 1B; Lizcano et al., 2012). The heterogeneity in the number and types of GTs encoded on the islands suggests significant glycan diversity decorating the glycoproteins. Indeed, mass-spectrometry revealed multiple glycoforms of the *S. agalactiae* SRRP Srr1 (Chaze et al., 2014). *L. reuteri* is also reported to display strain-specific glycosylation of its SRRP (Latousakis et al., 2019). The diversity and identity of glycan structures decorating many SRRPs are still unknown because extensive biochemical and analytical chemistry techniques are needed for such identification (Gloster, 2014).

Some streptococci encode multiple SRRPs while others lack SRRPs entirely. Approximately 50% of pneumococcal strains carry *psrP*, which is more likely to be found in strains isolated from individuals with pneumonia (Selva et al., 2012). *S. agalactiae* strains carry glycosylation islands encoding either Srr1 or Srr2, but not both at once, and the Srr1 island contains five accessory genes more than the Srr2 island (Figure 1B; Seifert et al., 2006). Genome-wide association studies revealed that *srr2* and associated GTs are almost exclusively found in hypervirulent strains of *S. agalactiae* (Seifert et al., 2006; Six et al., 2015; Gori et al., 2020). *S. gordonii* strains also express one of two SRRPs named GspB or Hsa. Unlike *S. agalactiae*, the glycosylation island associated with *gspB* and *hsa* are otherwise identical in content (Figure 1B; Bensing and Sullam, 2002; Takahashi et al., 2002). *S. salivarius* and *S. oralis* subsp. *dentisani* strains may carry a glycosylation island associated with three different SRRP alleles (Figure 1B; Couvigny et al., 2017; Ronis et al., 2019). Neither *Streptococcus pyogenes*, the causative agent of streptococcal pharyngitis and rheumatic fever, nor *Streptococcus mutans*, a significant contributor to dental caries, encode SRRPs (Mitchell, 2003).

While all SRRPs ultimately mediate adherence, variations in binding partners most likely contribute to the heterogenous colonization sites and disease of streptococcal pathogens. Polymorphisms of *srr1*, *srr2*, *gspB*, and *psrP* result in further variations of protein size and binding specificities (Samen et al., 2007; Zhou and Wu, 2009; Lizcano et al., 2012; Bensing et al., 2019). SRRPs from *S. pneumoniae* and *S. agalactiae* bind directly to the polypeptide backbone of keratin and fibrinogen (Samen et al., 2007; Shivshankar et al., 2009; Seo et al., 2013a). In contrast, SRRPs from oral streptococci tend to bind the glycan moiety, specifically those containing a terminal sialic acid, of glycoproteins such as platelet GPIbα (Bensing et al., 2004, 2016, 2019; Plummer et al., 2005; Singh et al., 2017; Ronis et al., 2019). The lectin-like properties of oral streptococcal SRRPs may permit binding to a larger variety of host structures but with lower affinity or specificity compared to SRRPs from *S. agalactiae* and *S. pneumoniae.*

## Biogenesis and Export of SRRPs

Prokaryotic protein glycosylation typically occurs in an *en bloc* fashion, where the glycan is assembled prior to transfer to a protein, or sequentially, where individual sugars are added to the growing chain on a polypeptide backbone (reviewed in Dell et al., 2010; Tan et al., 2015; Li et al., 2017; Schäffer and Messner, 2017). SRRPs are sequentially glycosylated in the cytoplasm prior to export (Chen et al., 2016, 2018; Zhu et al., 2016). Due to the diversity of accessory GTs encoded in individual glycosylation islands, it is not possible to generalize the pathways from any one species. To further add to the complexity of the glycosylation pathways, studies of *S. parasanguinis* revealed a novel bifunctional GT that sequentially adds two different monosaccharides to the growing glycan chain, and an *in vitro* study of *S. pneumoniae* suggests that multiple pneumococcal GTs can utilize different sugars to create polymorphic glycan decorations (Zhang et al., 2014, 2016; Jiang et al., 2017). The biogenesis of *S. gordonii* and *S. parasanguinis* SRRPs are best studied and have been reviewed in detail; comparatively little is known about *S. pneumoniae* and *S. agalactiae* (Zhou and Wu, 2009; Zhu et al., 2015; Zhu and Wu, 2016; Schäffer and Messner, 2018).

Regulated glycosylation is important for SRRP export and function. The first step in SRRP glycosylation is the covalent attachment of an *N-*acetylglucosamine (GlcNAc) moiety to the *O-*hydroxyl group of Ser or Thr residues on the SRR regions of the protein (*O*-linked glycosylation) by the cooperative action of GtfA and GtfB. With the exception of *S. parasanguinis*, mutation of *gtfA* or *gtfB* homologs leads to a complete loss of SRRP expression or aggregates of insoluble pre-protein in the cytoplasm (Takamatsu et al., 2004a; Wu et al., 2007; Mistou et al., 2009; Lizcano et al., 2017), thereby complicating attempts to dissect the role of glycosylation in SRRP activity. To bypass this problem, many studies either examine the BR in isolation or utilize a truncated version of the protein lacking most of the SRR2 region. While useful, such approaches preclude examination of the role that SRR2 plays in ligand binding and protein conformation.

The accessory secretion system is involved in maturation and transport of SRRP to the cell surface, upon which SRRPs are anchored to the cell wall by the housekeeping sortase (Nobbs et al., 2007; Mistou et al., 2009; Turner et al., 2009; Seepersaud et al., 2010, 2012; Yen et al., 2011). Some streptococcal strains encode additional Asp proteins (Asp4 and Asp5) that share homology with the secretion system components SecE or SecG, respectively (Takamatsu et al., 2005; Braunstein et al., 2019). Disruption of Asp2 prevents *O-*acetylation of the GlcNAc moiety, promoting hyperglycosylation and reducing binding of *S. gordonii* GspB to platelets (Seepersaud et al., 2012, 2017). Parallel work in *S. parasanguinis* supports the model that the accessory secretion system facilitates maturation of the glycoprotein (Wu et al., 2007; Li et al., 2008). The presence of *O-*acetylated GlcNAc in the mature *S. agalactiae* Srr1 coupled with cytoplasmic retention of hyperglycosylated forms of the protein further suggests that differential glycosylation regulates Srr1 secretion and function (Mistou et al., 2009; Chaze et al., 2014).

## Pneumococcal PsrP Facilitates Lung Colonization and Pneumonia

**FIGURE 2:**
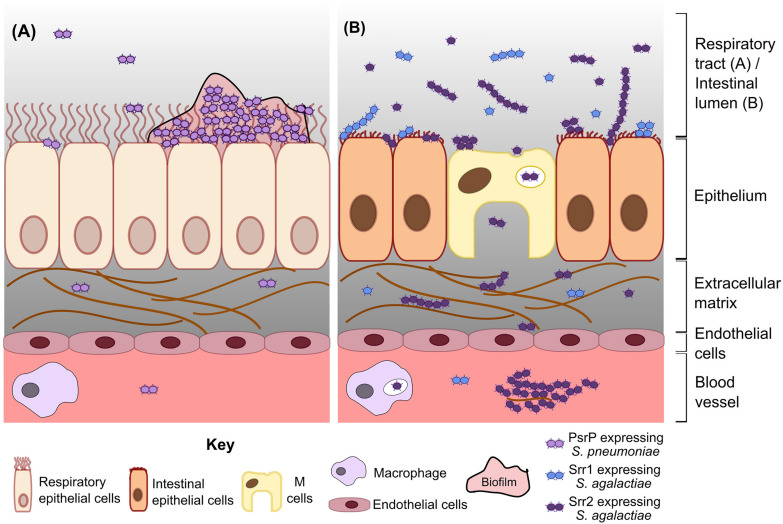
Model of *S. pneumoniae* and *S. agalactiae* SRRP functions in colonization and disease. **(A)** Pneumococcal PsrP mediates biofilm formation and persistent colonization in the respiratory tract, indirectly resulting in invasion of deeper tissue by a subset of bacteria. **(B)** Srr1 and Srr2 expressing *S. agalactiae* (blue and purple diplococci, respectively) adhere to intestinal epithelial cells during colonization. Srr2 promotes invasion to a greater extent than Srr1, partly by mediating bacterial adherence to and transcytosis through M cells. In the bloodstream, Srr2 facilitates persistence by forming large bacterial-plasma aggregates and by increasing survival of phagocytosed bacteria. Internalized *S. agalactiae* may exploit immune cell migration to disseminate and cause other diseases, e.g., meningitis.

The pneumococcal SRRP, PsrP, facilitates a non-inflammatory, persistent lifestyle in the nasopharynx and lungs (Figure 2A). PsrP has a multidomain BR that allows binding to keratin 10, fibrinogen, extracellular DNA, and other PsrP, supporting autoaggregation and biofilm formation (Rose et al., 2008; Shivshankar et al., 2009; Sanchez et al., 2010; Blanchette-Cain et al., 2013; Schulte et al., 2016). Mutation of *psrP* reduces bacterial load in the lungs and blood but not in the nasopharynx of intratracheally infected mice, partly because nasopharyngeal cells do not express keratin 10 (Rose et al., 2008; Shivshankar et al., 2009). Accordingly, immunization of mice against the BR of PsrP reduces pneumococcal burden in the lungs and blood of intranasally infected mice (Rose et al., 2008; Shivshankar et al., 2009). Mutation of *psrP* or *gtfAB* impairs biofilm formation and adhesion to lung cells, suggesting that glycosylation is crucial for PsrP function (Lizcano et al., 2017). Mutation of *psrP* also impairs biofilm formation in murine nasopharynx without altering bacterial numbers in this niche (Blanchette-Cain et al., 2013; Lizcano et al., 2017). Biofilm formation on murine nasal septa co-occurs with sloughing of ciliated cells and exposure of basement membranes, but loss of PsrP actually increases production of inflammatory cytokines such as IL-6 (Blanchette-Cain et al., 2013).

When pneumococci reach the lungs, likely mediated by other virulence factors, PsrP promotes biofilm formation and pneumonia. It is uncertain, however, whether PsrP directly mediates pneumococcal escape from the lungs into the bloodstream, or if invasion is an indirect consequence of high bacterial burden in lung biofilms. Mice infected intraperitoneally with wild type (WT) and *psrP* mutants show similar bacterial burden in the blood (Rose et al., 2008). This observation is partially explained by minimal *psrP* expression in murine blood (Shenoy et al., 2017). PsrP is also minimally expressed during planktonic growth but is significantly upregulated during stationary phase and biofilm formation (Lizcano et al., 2017). Indeed, pneumococci in biofilms are hyperadhesive and less invasive compared to the same strain grown planktonically (Blanchette-Cain et al., 2013), suggesting that PsrP may contribute to a non-invasive, hyperadhesive state that supports persistent colonization (Figure 2A). Further investigation of the regulation of PsrP may clarify its contribution to invasion and systemic disease.

## *Streptococcus agalactiae* Srr1 Promotes Adherence to Multiple Host Cell Types

The *S. agalactiae* SRRP, Srr1, binds fibrinogen and keratin 4, allowing *S. agalactiae* to colonize multiple body sites (Samen et al., 2007; Seo et al., 2012). Mutation of *srr1* impairs the ability of *S. agalactiae* to bind brain endothelial cells, laryngeal and lung epithelial cells, intestinal epithelial cells, vaginal and cervical cells, and platelets (Samen et al., 2007; Mistou et al., 2009; van Sorge et al., 2009; Sheen et al., 2011; Seo et al., 2013b; Wang et al., 2014). The ability of Srr1 to bind human platelets and brain endothelial cells is a direct consequence of its capacity to bind fibrinogen on their cell surface, while adherence to the vaginal and cervical epithelium is mediated by binding to both fibrinogen and keratin 4 (Sheen et al., 2011; Seo et al., 2012, 2013b; Wang et al., 2014). *S. agalactiae srr1* mutants are carried at lower density and duration compared to WT in a murine vaginal colonization model, suggesting that Srr1 promotes persistent vaginal colonization (Sheen et al., 2011; Wang et al., 2014). Mutation of *gtfAB* abolishes Srr1 surface expression, while deletion of all six accessory glycosyltransferases (Δ*gtfC-H*) results in a smaller glycoform of Srr1 (Mistou et al., 2009). The Δ*gtfC-H* mutant also displays increased trypsin sensitivity, resulting in loss of Srr1 on the cell surface and decreased adherence to lung and intestinal epithelial cells (Mistou et al., 2009). As such, glycosylation enhances Srr1 stability by resisting proteolytic inactivation, allowing for longer durations of adherence and persistence.

Mutations of *srr1* or associated glycosyltransferases attenuate *S. agalactiae* pathogenesis. When mice or rats are infected with a *srr1* mutant, the rodents show greater survival and lower bacterial counts in the brain and spleen, with fewer cardiac vegetations in an endocarditis model (Mistou et al., 2009; van Sorge et al., 2009; Seo et al., 2012, 2013b). Rat pups infected with the Δ*gtfC-H* mutant show improved survival compared to pups infected with WT *S. agalactiae*, emphasizing the importance of Srr1 glycosylation in pathogenesis (Mistou et al., 2009). Whether Srr1 promotes upper reproductive tract infections or enhances mother-to-child transmission are currently open questions. As mentioned earlier, Srr1 promotes persistent colonization of the vaginal tract and, while frequently asymptomatic, can lead to amnionitis or bacteremia in pregnant women (Seale et al., 2017). Additionally, carriage of *S. agalactiae* by pregnant women increases the risk of transmission and invasive *S. agalactiae* disease in the neonates (Seale et al., 2017). Expression of *srr1* requires the transcription factor Rga, which also regulates the pilin subunit *pilA* (Mistou et al., 2009; Samen et al., 2011). Future studies into the temporal and environmental regulation by Rga in *S. agalactiae* may provide greater insight into the role of Srr1 in health and disease.

## *Streptococcus agalactiae* Srr2 Is Associated With Increased Virulence and Meningitis

Expression of Srr2 enhances the ability of *S. agalactiae* to cause invasive disease, especially meningitis. The *srr2* allele is predominantly carried by hypervirulent strains, which are responsible for most cases of *S. agalactiae*-induced infant meningitis (Seifert et al., 2006). Srr2 facilitates binding to brain endothelial cells, vaginal, cervical and intestinal epithelial cells, and mutation of *srr2* reduces bacterial load in the brain, liver and mesenteric lymph nodes of infected mice (Sheen et al., 2011; Seo et al., 2013a; Wang et al., 2014; Six et al., 2015; Hays et al., 2019). Srr2 is expressed at higher levels and binds fibrinogen with higher affinity than Srr1, further enhancing the adherence ability of hypervirulent strains (Seo et al., 2013a; Six et al., 2015). Unlike *srr1*, strains carrying *srr2* do not encode a *rga* equivalent, and the regulator of Srr2 expression is unknown (Mistou et al., 2009). Nonetheless, Srr2 is expressed during invasive *S. agalactiae* disease; immunohistology from a fatal case demonstrated the presence of Srr2-expressing bacterial aggregates in the infant’s brain and liver (Six et al., 2015). Encouragingly, immunization of mice with recombinant Srr2 fragment protected 60–70% of mice infected intravenously with a LD_80_ dose of *S. agalactiae* (Six et al., 2015).

Expression of Srr2 promotes transcytosis through intestinal epithelium and dissemination to other body sites (Figure 2B). Srr2 facilitates binding to and transcytosis through murine intestinal M cells, leading to accumulation of the bacteria in mesenteric lymph nodes and translocation to the brain of mice orally gavaged with *S. agalactiae* (Hays et al., 2019). Increased adherence to intestinal cells is also likely to promote persistent colonization in the gastrointestinal tract. *S. agalactiae* expressing *srr2* forms large aggregates in the presence of plasma, which is likely mediated by the ability of Srr2 to bind the plasma proteins fibrinogen, plasminogen, and plasmin (Six et al., 2015). This aggregation increases internalization of the bacteria by macrophages and neutrophils (Six et al., 2015). However, Srr2 also promotes intracellular survival of *S. agalactiae*, potentially allowing a surviving population to hitch hike to distal sites or transmit from person to person (Six et al., 2015). In essence, Srr2 promotes bacterial migration out of the intestinal tract and to other organs, potentially by hijacking of immune cells.

## Discussion and Future Directions

As presented here, there is an emerging body of evidence that *S. pneumoniae* and *S. agalactiae* SRRPs facilitate adherence to mucosal surfaces, persistent infections and the transition to disease (Figure 2). In general, PsrP promotes persistent colonization and pneumonia through biofilm formation but is largely dispensable during systemic infections caused by *S. pneumoniae.* Meanwhile, Srr1 and Srr2 promote colonization and systemic disease such as meningitis by *S. agalactiae*. Invasiveness may be enhanced in hypervirulent *S. agalactiae* lineages through the increased binding affinity of Srr2 to plasma components, as well as the ability of Srr2 to mediate transcytosis through intestinal M cells and disseminate throughout the body by exploiting immune cells (Six et al., 2015; Hays et al., 2019).

Given their association with colonization and pathogenesis, SRRPs have emerged as potential vaccine targets. Passive and active immunization against PsrP BR and Srr2 N-terminal region ameliorate disease and reduce bacterial burden in infected animals, which are recapitulated in studies of the *S. aureus* SRRP SraP (Zhou et al., 2019). Administration of purified Srr1 and Srr2 BRs shortly before and after inoculation with *S. agalactiae* reduces bacterial burden in a murine vaginal colonization model, suggesting that blocking SRRP-mediated binding may prevent transmission (Sheen et al., 2011). However, the recombinant protein fragments used in these studies were generated in *Escherichia coli* strains and likely not glycosylated. This prompts the question of whether glycosylated forms of SRRP would elicit stronger immune responses. Antibodies generated against native SRRPs from *S. parasanguinis* and *S. gordonii* bind to both peptide and glycan components of the proteins (Bensing and Sullam, 2002; Stephenson et al., 2002; van Sorge et al., 2009). Additionally, highly opsonic antibodies generated during natural MRSA infections specifically recognize the glycosylated domain of the *S. aureus* glycoprotein ClfA (Hazenbos et al., 2013). In recent years, there has been remarkable advancement in engineering novel glycoconjugate vaccines, but much less effort is expended on adopting bacterial glycoproteins as vaccine targets.

Current pneumococcal anticapsular vaccines are effective in reducing invasive pneumococcal disease but are less effective in controlling colonization, particularly in high carriage burden settings (Swarthout et al., 2020). Given that passive immunization against PsrP reduces bacterial burden in murine lungs (Rose et al., 2008), we speculate that adding recombinant PsrP to the vaccine formulation, either as a conjugate protein or alongside the existing vaccines may improve efficacy in reducing pneumococcal carriage and non-invasive pneumonia. Vaccines for *S. agalactiae* are still in development, but adding Srr1 and Srr2 to a multivalent vaccine may also improve efficacy. It is recognized, however, that since SRRPs are not expressed by all *S. pneumoniae* and *S. agalactiae* isolates, an SRRP vaccine component cannot be expected to confer universal protection and therefore would need to be part of a multi-component or glycoconjugate vaccine. Further investigation into the distribution, regulation, and immunogenicity of SRRPs and other similar glycoproteins will better inform of their potential utility as vaccine candidates and may identify new carrier proteins for glycoconjugate vaccines.

## Author Contributions

JC and RH conceptualized the review. JC planned, wrote, and revised the manuscript. AG, AN, and RH critically read and revised the manuscript. All authors contributed to the article and approved the submitted version.

## Conflict of Interest

The authors declare that the research was conducted in the absence of any commercial or financial relationships that could be construed as a potential conflict of interest.
